# The role of early ezetimibe combination with atorvastatin in patients with atherosclerotic cardiovascular disease

**DOI:** 10.1186/s12872-026-05594-2

**Published:** 2026-02-11

**Authors:** Si-Hyuck Kang, Sung Uk Kwon, Jong-Young Lee, Suk Min Seo, Chang-Wook Nam, Gyung-Min Park, Young Joon Hong, Won Young Lee, Jung Eun Jang, In-Ho Chae

**Affiliations:** 1 Seoul National University Bundang Hospital, Seongnam-si, Korea; 2 Inje University College of Medicine, Ilsan Paik Hospital, Goyang, Korea; 3 Kangbuk Samsung Hospital, Sungkyunkwan University School of Medicine, Seoul, Korea; 4 Hallym University College of Medicine, Hallym University Sacred Heart Hospital, Anyang, Korea; 5 Eunpyeong St. Mary’s Hospital, College of Medicine, The Catholic University of Korea, Seoul, Korea; 6 Dongsan Hospital, Keimyung University, Daegu, Korea; 7 Ulsan University Hospital, University of Ulsan College of Medicine, Ulsan, Korea; 8 Chonnam National University Medical School, Chonnam National University Hospital, Gwangju, Korea; 9 Organon, Seoul, Korea

**Keywords:** Atherosclerotic cardiovascular disease, Atorvastatin, Early combination, Ezetimibe, Low-density lipoprotein-cholesterol goals, Very high-risk patients

## Abstract

**Background:**

Despite statin therapy, achieving target low-density lipoprotein cholesterol (LDL-C) levels remain suboptimal in high-risk patients with atherosclerotic cardiovascular disease (ASCVD). This study evaluated efficacy and safety of early addition of ezetimibe (EZ) with atorvastatin (AS), prior to reaching the maximally tolerated dose of statin, in very high-risk patients.

**Methods:**

This phase 4 (NCT05761444), multicenter, randomized, open-label, active-controlled study enrolled patients (≥ 30 years) with very high-risk of ASCVD. Eligible patients had LDL-C ≥ 70 mg/dL with low/moderate intensity statin monotherapy or statin-naïve or not been on stable statin regimen prior to enrollment. Patients were randomized 1:1 to EZ10/AS40 mg combination therapy or AS40 mg statin alone for 12 weeks. Primary endpoint was percentage change in LDL-C from baseline to week 6.

**Results:**

Patients (*N* = 137) received EZ/AS (*n* = 67) or AS (*n* = 70) once a day. The EZ/AS lipid-lowering effect was statistically greater than AS monotherapy at week 6 (LSMD: ˗21.2; *P* < 0.0001) and week 12 (LSMD: ˗16.0; *P* < 0.0001). At week 12, higher proportions of patients who received EZ/AS achieved target LDL-C < 55 mg/dL (55.0% vs. 15.4%; *P* < 0.0001) and LDL-C < 70 mg/dL (85.0% vs. 58.5%; *P* = 0.0009) than in AS group. Higher reduction from baseline was observed for lipid parameters in EZ/AS group than AS monotherapy. Incidence of adverse events were comparable between EZ/AS and AS groups.

**Conclusions:**

Early combination of EZ with AS, rather than a stepwise approach, significantly reduced LDL-C levels and improved LDL-C reduction target achievement compared to AS monotherapy in very high-risk patients with dyslipidemia with no new safety issues.

**Trial registration:**

NCT05761444; Registration date: March 9, 2023.

**Supplementary Information:**

The online version contains supplementary material available at 10.1186/s12872-026-05594-2.

## Introduction

Cardiovascular disease (CVD) remains a leading cause of morbidity and mortality worldwide, with dyslipidemia being a major modifiable risk factor along with smoking, hypertension, diabetes, and obesity [1, 2]. Managing blood lipids is crucial for these patients. Dyslipidemia treatment includes non-drug therapy and drug therapies [3]. Low-density lipoprotein cholesterol (LDL-C) contributes to the development of atherosclerotic cardiovascular disease (ASCVD), and reducing LDL-C levels is linked to lowering CV risk [4]. Statins are the cornerstone of lipid-lowering therapy (LLT), but many patients need additional LDL-C reduction beyond what can be achieved with statin monotherapy [5]. High-intensity statin therapy is often limited by adverse effects, particularly in Asian populations, who may have heightened sensitivity due to genetic polymorphisms [6, 7]. Given these considerations, non-statin therapies play an important role in achieving recommended lipid targets.

The 2025 European Society of Cardiology/European Atherosclerosis Society (ESC/EAS) guidelines recommend aggressive LDL-C lowering, with LDL-C goal of < 55 mg/dL (< 1.4 mmol/L) for very high-risk patients and < 70 mg/dL (< 1.8 mmol/L) for high-risk patients with ASCVD [8]. These guidelines recommend early initiation of combination LLT, including the immediate initiation of statin therapy along with ≥ 1 classes of non-statin therapy with proven CV benefits, such as ezetimibe (EZ) or bempedoic acid, taken alone or in combination, when LDL-C goals are not achieved with the maximum tolerated statin dose. Early, intensive LDL-C lowering is recommended for all acute coronary syndrome (ACS) patients, with therapy tailored to prior LLT. The choice of therapy should depend on the magnitude of additional LDL-C reduction, patient preference, treatment availability, and cost [8]. Similarly, the recently updated ACC/AHA 2025 guidelines for ACS also support adding non-statin LLT if LDL-C remains above 55 mg/dL [9]. Collectively, these guidelines emphasize the importance of early combination therapy in achieving LDL-C targets.

Ezetimibe, when combined with statin therapy, lowers LDL-C by inhibiting cholesterol reabsorption from bile in the liver and in the small intestine [10]. The IMPROVE-IT study demonstrated that adding EZ to simvastatin provided incremental CV benefit in patients with acute coronary syndrome, particularly in specific high-risk subgroups [11]. Studies have shown that EZ in combination with atorvastatin (AS) achieved significantly greater reduction in LDL-C, total cholesterol, and triglyceride reductions compared to escalating AS doses. This combination (EZ and AS) was well tolerated and had a safety profile similar to statin monotherapy [12]. Moreover, the EWTOPIA 75 study showed that LDL-C lowering therapy with EZ prevented CV events, when used as primary prevention among individuals aged 75 years or more with elevated LDL-C levels [13].

Recent evidence suggests that early initiation of combination therapy may be more effective in achieving early and sustained LDL-C control than initiating after statin failure [14]. A recent study from the SWEDEHEART registry showed that early combination of EZ to high-intensity statin was associated with greater benefits in reducing major adverse cardiovascular events compared to delayed combination therapy in LLT in naïve patients hospitalized for myocardial infarction [15].

This concept of early combination therapy is a significant shift in dyslipidemia management, particularly for patients with ASCVD who need intensive lipid-lowering to achieve target LDL-C levels. Observational studies have reported that approximately one third of patients with myocardial infarction are already on LLT, mostly low- to moderate-intensity [16, 17]. For those who are already on LLT, % LDL-C reduction from baseline is not feasible and LLT is intensified based on target LDL-C. While contemporary guidelines recommend a stepwise approach, high-intensity statin is usually not enough to achieve LDL-C < 55 mg/dL. Addition of ezetimibe to statin has been shown to be a cost-effective way for secondary prevention [18]. Several studies have evaluated the efficacy of combination therapy in Western populations [19]; However, there is limited data specifically addressing the efficacy and safety of early EZ-statin combination therapy in patients with ASCVD.

## Methods

### Study design

This phase 4, multicenter, randomized, open-label, active-controlled study evaluated the efficacy and safety of early add-on of EZ with AS in patients with ASCVD and LDL-C above 70 mg/dL. The study included a screening period (0–1 week) for patients with very high-risk and a treatment (intervention) period (12 weeks). Patients were eligible if their LDL-C was above 70 mg/dL with low to moderate intensity statin monotherapy; or were statin-naïve or had not been on a stable statin regimen for at least 4 weeks prior to enrollment. All laboratory tests were performed in the local laboratory. Eligible patients were randomized 1:1 to receive either EZ/AS combination therapy or AS monotherapy orally once a day.

Patients who reached the < 55 mg/dL LDL-C target at 6 weeks continued at the same dose for 6 more weeks as assigned. If the LDL-C target was not reached, the dose of AS was increased to EZ10/AS80 mg or AS80 mg accordingly for the remaining 6 weeks. At 12 weeks, efficacy and safety were assessed (Supplementary Fig. 1).

### Study oversight

This study was conducted in compliance with all applicable regulatory requirements, the International Council for Harmonization, Good Clinical Practice, and in accordance with the principles of the Declaration of Helsinki. The study protocol was approved by the institutional review board of each site, and written informed consent was obtained from all participants before commencing any study-specific procedure. The study is registered with clinicaltrials.gov (NCT05761444).

### Patients

Patients ≥ 30 years with very high-risk of ASCVD (clinical or unequivocal on imaging ASCVD) and had LDL-C 70 mg/dL or higher were eligible for the study. ASCVD included myocardial infarction (MI), unstable/stable angina, coronary revascularization, coronary bypass graft surgery, other arterial revascularization procedures, stroke, transient ischemic attack, and peripheral arterial disease. Patients with LDL-C levels ≥ 70 mg/dL, who were willing to maintain a therapeutic lifestyle change throughout the study and those who provided the written informed consent prior to study enrollment were included.

The following were the major exclusion criteria for this study: Hypersensitivity to EZ, AS or any of its inactive ingredients; Active liver disease or aspartate transaminase (AST) or alanine transaminase (ALT) >3x upper limit of normal; Pregnant woman or intending to become pregnant within a year; History of cancer within 5 years; Patients with disease known to influence serum lipids or lipoproteins excluding dyslipidemia.

### Efficacy and safety outcomes

The primary endpoint was percentage change in LDL-C from baseline to week 6. The secondary endpoints were proportion of patients achieving LDL-C goal of < 55 mg/dL after 6 weeks and 12 weeks, proportion of patients achieving LDL-C goal of < 70 mg/dL after 6 weeks and 12 weeks, percentage change in LDL-C from baseline to week 12, percentage change in high-density lipoprotein cholesterol (HDL-C), non-HDL-C, triglycerides, and total cholesterol from baseline to week 6 and week 12.

Safety was assessed based on the number and percentage of patients experiencing any treatment-emergent adverse events (TEAEs), treatment-emergent serious adverse events (TESAEs), treatment-related adverse events (TRAEs) and TEAEs leading to the premature discontinuation of the study at 6 weeks and 12 weeks.

### Statistical analyses

The safety analysis set (SAS) included all randomized patients who received at least one dose of study intervention. Patients in the SAS were included in the actual intervention group, regardless of their randomized treatment. The full analysis set (FAS) comprised of all randomized patients who received at least one dose of study treatment and had a baseline assessment of LDL-C and at least one valid post baseline measurement of LDL-C. Patients in the FAS were counted towards their randomized intervention group, regardless of their actual treatment. The FAS is the primary analysis set, which is used for efficacy analyses. All analyses were performed by a designated CRO (Clinical Research Organization) using SAS 9.4 or above.

For continuous efficacy variables, the statistical comparisons between intervention groups were performed using an analysis of covariance (ANCOVA) with treatment group as a factor and the corresponding baseline and history of statin administration as the covariate. Estimates of least squares (LS) mean and standard error (SE) for within-treatment effects and treatment differences along with corresponding two-sided 95% CI and P-value are provided. If the upper limit of the 95% two-sided CI for the corresponding treatment difference (EZ10/AS40 mg - AS40 mg) was less than 0, EZ10/AS40 mg was considered superior to AS40 mg. Sensitivity analysis regarding missing efficacy parameters was performed to assess the robustness of the primary endpoint results.

For categorical efficacy variables, the statistical significance of the differences between treatment groups was tested using Chi-square test or Fisher’s exact test. The difference in the percentage was presented with corresponding 95% CI and P-value. The numbers and percentages of patients experiencing TEAEs were presented by system organ class (SOC) and preferred term (PT) according to the MedDRA dictionary for each treatment group.

All the statistical tests were performed with a two-sided α of 0.05. No imputation was performed for the primary analysis. For the sensitivity analysis of LDL-C, missing postbaseline LDL-C data for Week 6 were imputed by the last observation carried forward (LOCF) method based on the last valid post-baseline measurement prior to the visit. For patients with no post-baseline value, the baseline values were carried forward. All safety analyses were performed on the SAS.

## Results

### Patients

**Fig. 1:**
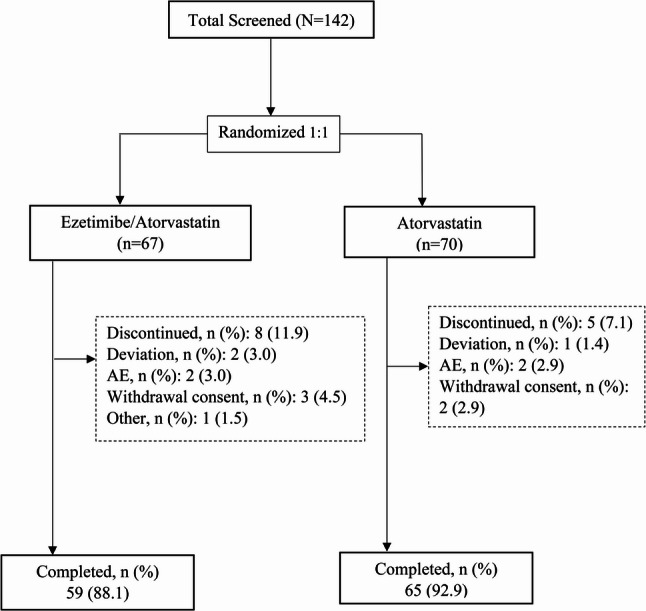
Patient disposition. AE=adverse event; N=number of patients in Full Analysis Set; n=number of patients in each category

Overall, 142 patients were screened, and 5 patients failed the screen due to violation of inclusion/exclusion criteria. Nearly two-thirds had a history of low/moderate intensity statin monotherapy. A total of 137 patients aged ≥ 30 years were randomized 1:1 to EZ/AS group (*n* = 67) and AS group (*n* = 70). Among these, 88.1% patients in EZ/AS group and 92.9% patients in the AS group completed the study (Fig. 1). Overall, 11.9% in EZ/AS group and 7.1% in the AS group patients discontinued the study. The most common reasons for discontinuation were consent withdrawal (EZ/AS: *n* = 3, 4.5%; AS: *n* = 2, 2.9%) and AEs (EZ/AS: *n* = 2, 3.0%; AS: *n* = 2, 2.9%). The baseline demographic and clinical characteristics were similar between the groups (Table 1). The average age of the patients was 65.0 ± 10.4 years, 54.5% were aged ≥ 65 years, and 78.0% were male. Mean body mass index was 24.9 ± 3.1 kg/m^2^. Overall, 25.8% of the patients were current smokers and 25.8% consumed alcohol. Mean ± SD of LDL-C level at baseline was 98.5 ± 24.0 mg/dL for EZ/AS intervention and 108.5 ± 34.4 mg/dL for AS. Baseline plasma lipid parameters were comparable between groups, both overall and when classified according to history of statin administration (Supplementary Table 1).

**Table 1:** Patient baseline demographic and clinical characteristics in Korea

Characteristics	EZ/AS (*N* = 65)	AS (*N* = 67)	Total (*N* = 132)
Age, y	65.1 (10.0)	64.9 (10.7)	65.0 (10.4)
< 65 y	26 (40.0)	34 (50.7)	60 (45.5)
≥ 65 y	39 (60.0)	33 (49.3)	72 (54.5)
Male	53 (81.5)	50 (74.6)	103 (78.0)
BMI, kg/m^2^	24.7 (3.0)	25.0 (3.2)	24.9 (3.1)
Smoking History			
Currently Smoking	19 (29.2)	15 (22.4)	34 (25.8)
Past Smoker	19 (29.2)	16 (23.9)	35 (26.5)
Never Smoked	27 (41.5)	36 (53.7)	63 (47.7)
Alcohol History			
Currently Drinking	14 (21.5)	20 (29.9)	34 (25.8)
Past Drinking	16 (24.6)	8 (11.9)	24 (18.2)
Never Drinking	35 (53.8)	39 (58.2)	74 (56.1)
Lipid profile, mg/dL			
LDL-C	98.5 ± 24.0	108.5 ± 34.4	103.6 ± 30.0
HDL-C	47.9 ± 12.9	49.6 ± 11.4	48.7 ± 12.1
non-HDL-C	114.5 ± 26.9	125.6 ± 38.1	120.0 ± 33.3
Triglycerides	131.6 ± 84.1	145.7 ± 85.6	138.8 ± 84.8
Total Cholesterol	162.4 ± 26.2	175.2 ± 38.9	168.8 ± 33.6

Values are mean ± SD or n (%). The analysis was based on the full analysis set

*AS* atorvastatin, *BMI* body mass index, *EZ* ezetimibe, *HDL-C* high-density lipoprotein cholesterol, *LDL-C* low-density lipoprotein cholesterol, *SD* standard deviation

BMI (kg/m^2^) = weight (kg)/ height (m)^2^

The mean ± SD duration of dyslipidemia was 4.2 ± 5.8 years. Among the patients, 65.2% had prior low and/or moderate intensity statin monotherapy for ≥ 4 weeks before the intervention (EZ/AS: 69.2% and AS: 61.2%) (Supplementary Table 2). The mean of intervention exposure was 79.6 ± 16.3 days, and comparable between the intervention and control groups. Compliance with the study intervention regimen was similar and high across the groups, with most participants maintaining > 95% adherence with the protocol-defined regimen (Supplementary Table 3).

### Efficacy outcomes

**Fig. 2:**
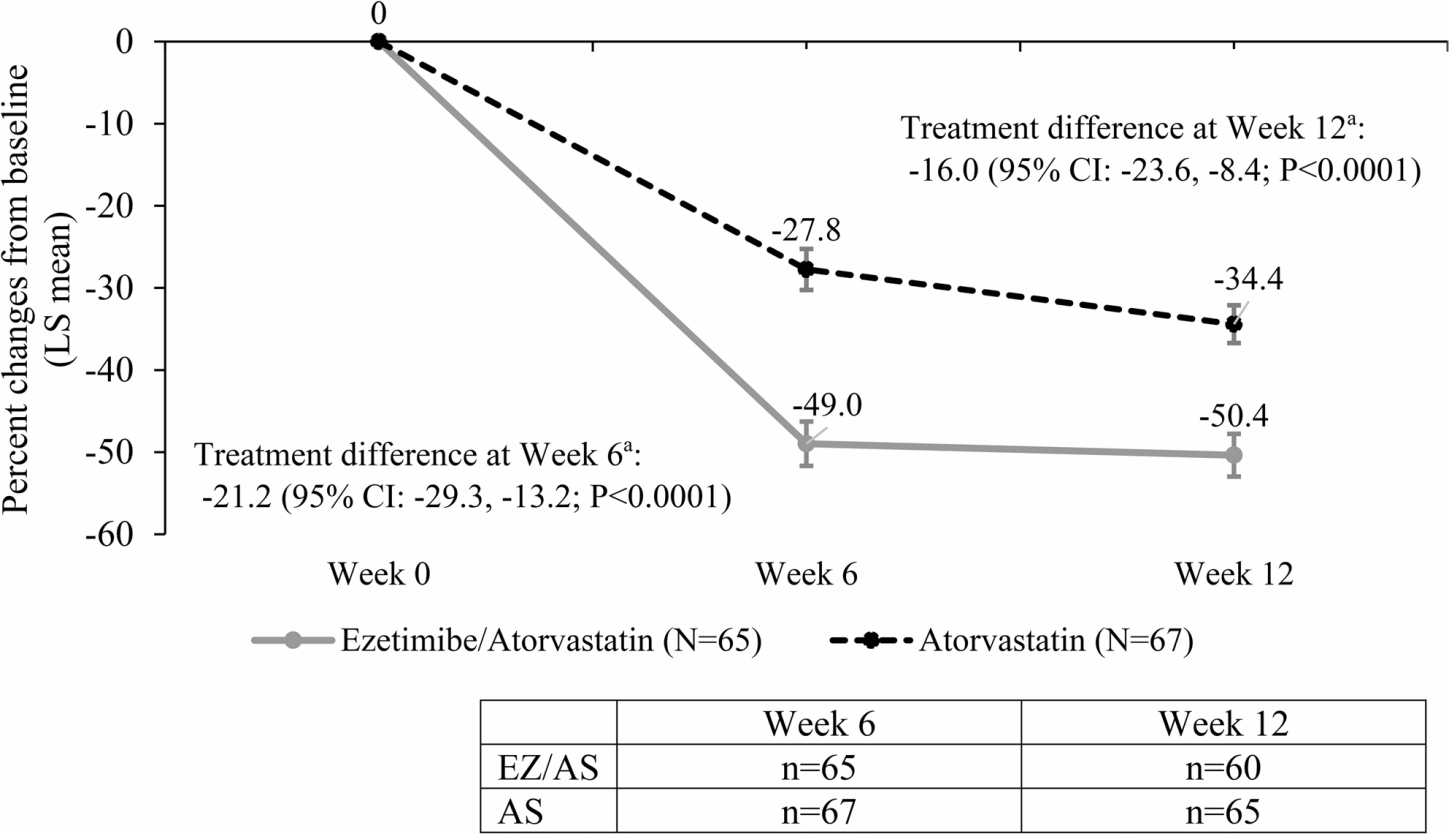
Percentage Changes in LDL-C After 6 and 12 Weeks of Intervention with EZ/AS or AS. aLS means, LS mean difference, and 95% CI for the LS mean difference from an ANCOVA model with treatment group (EZ/AS, AS) as factor and history of statin administration (Yes, No), and baseline LDL-C as covariates. AS=Atorvastatin; EZ=Ezetimibe/Atorvastatin; LDL-C=low-density lipoprotein cholesterol; N=number of patients in Full Analysis Set; n=number of patients without missing values

The treatment with EZ/AS combination therapy resulted in statistically greater and clinically meaningful lipid-lowering effect as compared with AS monotherapy at week 6 and was maintained through 12 weeks. (Fig. 2). The mean ± SD of LDL-C level at Week 6 was 58.4 ± 16.6 mg/dL for EZ/AS and 75.6 ± 18.5 mg/dL for AS group and at Week 12: EZ/AS = 54.8 ± 15.2 mg/dL, AS = 70.1 ± 16.7 mg/dL. The mean LDL-C levels from a sensitivity analysis, in which the missing post-baseline LDL-C data for Week 6 were imputed by LOCF based on the last valid post-baseline measurement prior to the visit, were similar to the primary analysis supporting the primary analysis results.

**Fig. 3:**
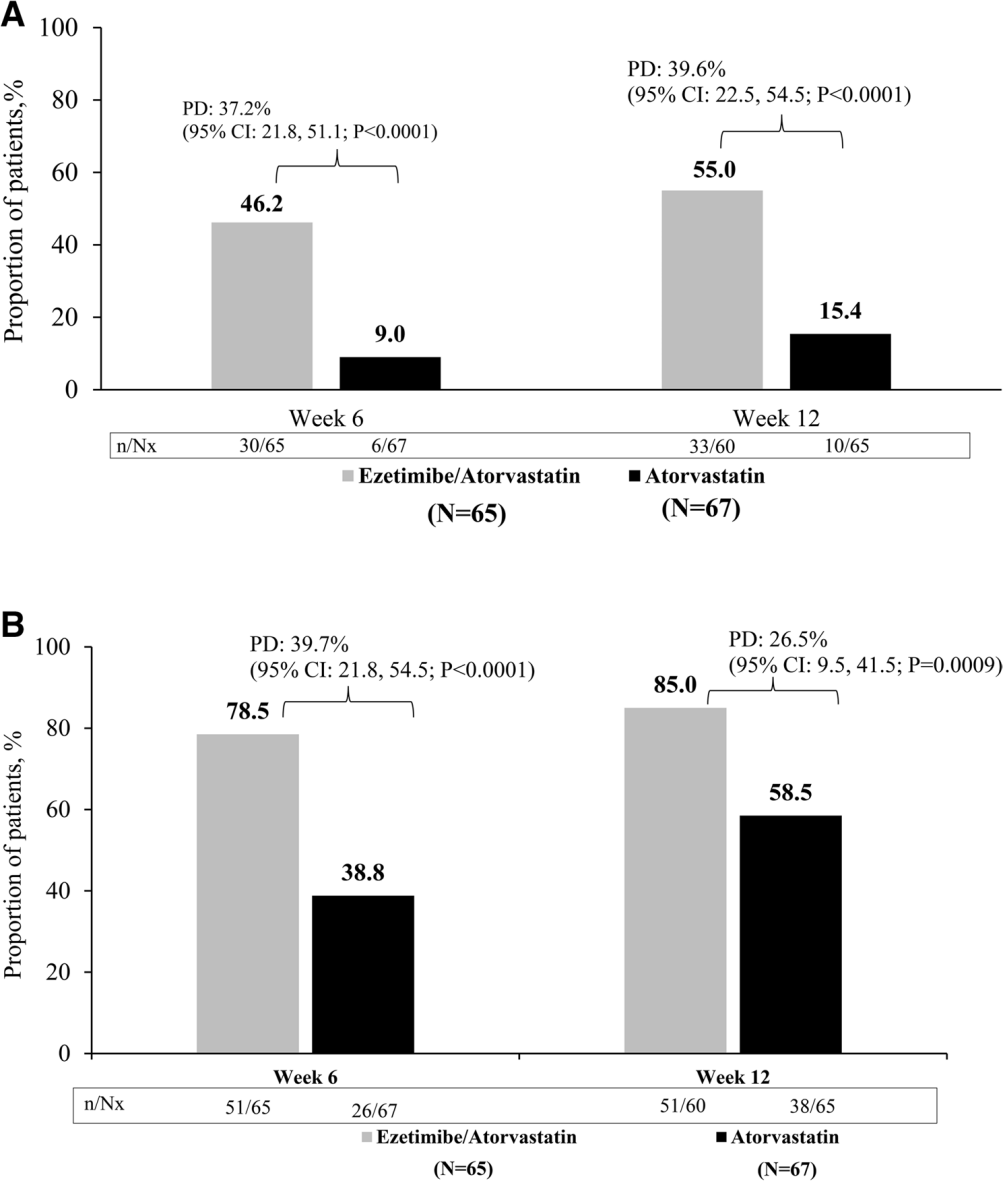
Achievement of LDL-C Targets at Week 6 and 12. **A**. Achievement of LDL-C level <55 mg/dL. **B**. Achievement of LDL-C level <70 mg/dL. CI=confidence interval; LDL-C=low-density lipoprotein cholesterol; N=number of patients in Full Analysis Set; Nx=number of patients without missing values; n=the number of patients achieving; PD=percentage difference.

A significantly higher proportion of patients achieved the target LDL-C levels of < 55 mg/dL in EZ/AS group compared with AS group at week 6 (46.2% vs. 9.0%, percentage difference [PD]: 37.2% [95% CI]: 21.8, 51.1; *P* < 0.0001) and at week 12 (55.0% vs. 15.4%, PD: 39.6% [95% CI]: 22.5, 54.5; *P* < 0.0001) (Fig. 3a). The target LDL-C level of < 70 mg/dL was achieved in greater proportion of patients in the EZ/AS group at week 6 (78.5% vs. 38.8%; PD: 39.7% [95% CI: 21.8, 54.5]; *P* < 0.0001) and at week 12 (85.0% vs. 58.5%, PD: 26.5% [95% CI: 9.5, 41.5]; *P* = 0.0009) as compared with AS monotherapy group (Fig. 3b).

The percentage change from baseline to week 12 for the lipid parameters were as follows: HDL-C (LS mean difference [LSMD] [95% CI]: ˗3.0 [˗10.6, 4.6]; *P* = 0.4390), non-HDL-C (LSMD [95% CI]: ˗10.2 [˗21.5, 1.1]; *P* = 0.0753), total cholesterol (LSMD [95% CI]: ˗8.1 [˗17.5, 1.3]; *P* = 0.0888), and triglycerides (Difference in medians [95% CI]: -8.0 [-20.3, 4.3]). A higher reduction in LS mean from baseline was noted with EZ/AS combination therapy than AS monotherapy for all the lipid parameters at week 6 and the similar effect continued till week 12 (Table 2).

**Table 2:** Percent change from baseline to week 6 and week 12 in lipid profiles

	EZ/AS (*N* = 65)	AS (*N* = 67)	LS mean difference (95% CI); *P*-value
Percent change from baseline to Week 6			
LDL-C	-49.0 (-54.4, -43.6)	-27.8 (-32.7, -22.8)	-21.2 (-29.2, -13.2); *P* < 0.0001
HDL-C	-3.9 (-9.5, 1.7)	-0.51 (-5.8, 4.8)	-3.4 (-10.9, 4.1); *P* = 0.3712
Non-HDL-C	-35.6 (-42.8, -28.4)	-29.3 (-36.0, -22.6)	-6.3 (-18.3, 5.8); *P* = 0.2975
Total Cholesterol	-28.6 (-33.3, -23.9)	-18.7 (-23.0, -14.4)	-9.9 (-17.3, -2.4); *P* = 0.0112
Triglycerides	-9.7 ± 40.8	-4.9 ± 59.6	-0.3 (-13.2, 12.5)^#^
Percent change from baseline to Week 12			
LDL-C	-50.4 (-55.6, -45.2)	-34.4 (-39.1, -29.8)	-16.0 (-23.6, -8.4); *P* < 0.0001
HDL-C	-3.7 (-9.4, 2.0)	-0.7 (-5.8, 4.4)	-3.0 (-10.6, 4.6); *P* = 0.4390
Non-HDL-C	-39.9 (-46.8, -33.1)	-29.7 (-35.8, -23.7)	-10.2 (-21.5, 1.1); *P* = 0.0753
Total Cholesterol	-29.9 (-35.9, -23.9)	-21.8 (-27.0, -16.5)	-8.1 (-17.5, 1.3); *P* = 0.0888
Triglycerides	-16.7 ± 31.9	-1.5 ± 65.6	-8.0 (-20.3, 4.3)^#^

Values are least squares mean (95% CI) except triglycerides are mean ± SD and difference in medians (95% CI)

#Estimate of difference in median location using the Hodges Lehmann method and corresponding 95% CI

The analysis was based on the full analysis set

*AS* atorvastatin, *CI* confidence interval, *EZ* ezetimibe, *HDL-C* high-density lipoprotein cholesterol, *LDL-C* low-density lipoprotein cholesterol, *LS* least squares, *N* number of patients in Full Analysis Set, *SD* standard deviation

### Safety

#### Adverse events

Safety analysis demonstrated similar adverse events (AEs) profiles for both groups at week 6 and week 12 (Table 3). TEAEs were reported in 28 (20.4%) patients (EZ/AS = 15; AS = 13) at week 12. In the EZ/AS group, 3 patients experienced AEs and discontinued treatment: 1 (1.5%) patient due to fatigue, 1 (1.5%) patient acute myocardial infarction (MI) and 1 (1.5%) patient dizziness. In the AS group, 4 patients discontinued treatment: 1 (1.4%) due to malignant stomach neoplasm and 3 (4.3%) patients due to myalgia.

**Table 3:** Summary of adverse events

Type of AEs	EZ/AS (*N* = 67)	AS (*N* = 69)	*P*-value^a^
At Week 12			
Any AE	15 (22.4)	13 (18.8)	0.3823
Any SAE	4 (6.0)	3 (4.3)	0.4835
Any TRAE	2 (3.0)	1 (1.4)	0.4889
Any AE leading to premature discontinuation	3 (4.5)	2 (2.9)	0.4860
At Week 6			
Any AE	9 (13.4)	8 (11.6)	0.4739
Any SAE	3 (4.5)	2 (2.9)	0.4860
Any TRAE	1 (1.5)	0	0.4926
Any AE leading to premature discontinuation	2 (3.0)	2 (2.9)	0.6791

Values presented as *n* (%)

^a^*P*-Values from a Fisher’s exact test

AS=atorvastatin; AE=adverse events; EZ=ezetimibe; N=number of patients in Safety Analysis Set; n=number of patients in each category; n = the number of patients in each category; percentage (%) = 100*(n/N); SAE=serious adverse event; TRAE=treatment-related adverse event deemed by the investigator to likely be caused by the intervention

Seven (5.1%) patients experienced serious AEs; however, investigators did not consider any of these to be related to the study interventions (Supplementary Table 4). AEs reported in > 2% of patients in any treatment group were ALT increased (3 patients, 2.2%) and dizziness (3 patients, 2.2%). No deaths occurred during the study period.

## Discussion

Early addition of EZ to AS, prior to reaching the maximally tolerated statin dose, enabled significantly more very high-risk Korean patients to achieve LDL-C goal of < 55 mg/dL and < 70 mg/dL compared to statin monotherapy, with a comparable safety profile in our study. The combination therapy addressed an important unmet need in the management of dyslipidemia at 6 weeks and maintained through 12 weeks in this study. The overall safety of EZ/AS combination therapy was consistent with the established safety profiles of EZ and AS alone.

The improved lipid-lowering efficacy of the EZ/AS combination compared to AS monotherapy observed in our study is consistent with previous findings which indicated that adding EZ to AS40 mg was significantly more effective than up titration to AS80 mg for lowering LDL-C levels [20]. The EXPLORER study assessed the efficacy of combination therapy of rosuvastatin (RS) 40 mg and EZ10 mg in very high-risk patients [21]. The combination group achieved a mean LDL-C level of 56.9 mg/dL at 6 weeks, which was significantly lower than 81.5 mg/dL in the RS40 mg monotherapy group [22]. The achieved LDL-C levels are numerically similar to those observed in our study (54.8 ± 15.2 mg/dL and 70.1 mg/dL, respectively). Given the use of high intensity statins such as RS40 mg is restricted due to an increased risk of adverse events in Asian populations, this study supports the advantages of using EZ10/AS80 mg in very high-risk patients.

A systematic review and meta-analysis of 20 randomized controlled trials (RCTs) with 5,412 participants found that low/moderate-intensity statin + EZ achieved greater LDL-C reduction than high-intensity statin monotherapy. Importantly, there was no significant difference in major adverse cardiovascular events (MACE), suggesting comparable safety and efficacy [23]. A large-scale meta-analysis of 14 studies (11 RCTs and 3 cohort studies) involving over 108,000 very high-risk patients showed that statin + EZ combination therapy significantly improved lipid profiles and CV outcomes compared to statin monotherapy. This supports broader use of combination therapy in high-risk populations [24].

Recent guidelines strongly recommend achieving LDL-C levels < 55 mg/dL in patients at very high-risk for CV events [8, 9]. Our findings are relevant for addressing the unmet need for lipid management. A survey of the Korean Society of Myocardial Infarction found that most clinicians were aware of the new treatment guidelines for high-risk patients; however, less than half of the clinicians applied LDL-C target goal of < 55 mg/dL in their practice. The most common reasons of not following the goal were an apparent lack of efficacy and safety data in Korean patients and concerns about the cost-effectiveness [20]. The DYSIS study worldwide demonstrated that only 31% of very high-risk patients and 46% of high-risk patients on statin therapy achieve recommended LDL-C goals [25]. Factors contributing to this treatment gap include insufficient high-intensity statin use, concerns about intolerance, and limited combination therapy use [26]. Therapeutic inertia is another hurdle. A non-interventional study revealed low LDL-C goal achievement and sub-optimal LLT use despite high treatment adherence [27]. Clinicians’ excessive caution in up-titrating LLT may hinder optimal use of combination treatments, and better adherence to guidelines could significantly enhance dyslipidemia management. The current study also showed that no greater than 9% and 15% (week 6 and 12, respectively) of patients achieved the target LDL-C < 55 despite the use of high-intensity statin. Addition of EZ early on significantly improved target LDL-C achievement rate to 46% and 55%. These results highlight the challenge of achieving optimal LDL-C control with high-dose statin monotherapy and underscore the value of combination therapy.

The 2023 ESC guidelines recommend high-intensity statin and EZ combination therapy during ACS hospitalization if patients have LDL-C levels that are unlikely to be controlled by statin alone [28]. The 2025 ACC/AHA guideline for ACS suggests that high-intensity statin therapy is recommended for all patients with ACS, and with an option to initiate EZ simultaneously. Addition of EZ is recommended for patients already on maximally tolerated statin with LDL-C ≥ 70 mg/dL (1.8 mmol/L). While, in high-risk patients, with LDL-C level: 55 to < 70 mg/dL (1.4 to < 1.8 mmol/L) and already on a maximally tolerated statin, intensification of LLT is recommended [9].

A prospective cohort study showed early combination therapy with atorvastatin 80 mg and EZ on admission achieved LDL-C target of < 55 mg/dL in 80% of patients with ST-elevation myocardial infarction after 4–6 weeks of discharge [29]. Evidence also suggests clinical benefits of early combination therapy in long-term outcomes. Lewek et al., analyzed the Polish ACS registry to find that upfront combination therapy (EZ and statin) compared to statin monotherapy (AS or RS) was linked to a reduction in all-cause mortality after propensity score matching [30].

The SWEDEHEART study assessed the impact of delayed treatment escalation on outcomes by comparing early (EZ added to statins ≤ 12 weeks after discharge) vs. late (13 weeks to 16 months) combination therapy in patients with MI. One-year MACE incidences were 1.79 (early), 2.58 (late), and 4.03 (none) per 100 patient-years. Compared with early combination therapy, weighted risk differences in MACE for late combination therapy at 1, 2, and 3 years were 0.6% (*P* < 0.01), 1.1% (*P* < 0.01), and 0.7% (*P* = 0.18) [15].

Proprotein Convertase Subtilisin/Kexin Type 9 (PCSK9) inhibitors are another option of non-statin LLT. A pharmacoeconomic evaluation from China highlighted that while PCSK9 inhibitors significantly reduce CV events, their high-cost limits routine use, especially when compared to statins and EZ [31]. Although previously limited by high cost and injectable administration, the cost has decreased and accessibility has improved in recent years. This has slightly improved prescribing patterns and continuation rates in eligible patients, despite persistent barriers to broader adoption [32, 33]. While EZ, particularly post-patent expiration, is seen as a more affordable alternative [31]. The availability of single-pill combinations, such as statin with EZ, improves adherence and accessibility, making them a practical choice in dyslipidemia management [34].

Emerging evidence also supports the possibility of early initiation of triple combination therapy for rapid LDL-C reduction after ACS. The LAI-REACT study in statin-naïve patients with ACS showed that a novel triple combination therapy (10 mg ezetimibe, 40 mg rosuvastatin, and 180 mg bempedoic acid daily) demonstrated significant LDL-C reduction within the first week, which was sustained through six weeks. This cost-effective regimen offers an accessible alternative to PCSK9 inhibitors [35].

In our study, EZ/AS therapy led to greater reductions in lipid profile as compared to AS monotherapy. The present study provides strong evidence that the addition of EZ to AS may help patients at very high-risk of CVD achieve significant improvements in their lipid levels. AE incidences were low in both the treatment groups, with no drug-related deaths reported in this study, which is consistent with previous studies demonstrating the favorable safety profile of combination therapy with EZ [20, 36].

### Study limitations

There were several limitations to this study. This was a small-sized trial with short-term clinical follow-up. The design and scope of this study were not intended to evaluate cardiovascular outcomes (e.g., death, myocardial infarction, or etc.). While evidence from cohort studies implies benefits of early combination therapy on clinical outcomes, these findings need to be validated in future large-scale randomized controlled clinical trials. Due to the open-label nature of the study, there was a potential to introduce bias. These findings underscore the importance of conducting larger, multinational studies in the future to further validate and expand upon the current results.

## Conclusions

The early addition of EZ to AS showed a significantly greater and clinically meaningful lipid-lowering effect in patients through week 12 compared to AS monotherapy. A higher proportion of patients achieved target LDL-C levels (< 55 mg/dL and < 70 mg/dL) with the EZ/AS combination than with AS alone. The combination of EZ and AS significantly improved LDL-C levels and other lipid parameters in a clinically meaningful way, with no new safety concerns. Therefore, this study implies that an initial combination therapy with EZ and statin, rather than a stepwise approach, may be considered for very high-risk patients with dyslipidemia.

## Supplementary Information

Supplementary Material 1.

## Data Availability

The subgroup analysis datasets generated and/or analyzed during the current study can be requested from the Organon Korea MAOR team at [krmaor@organon.com](mailto: krmaor@organon.com) .

## Declarations

**Table:**

Name of the Ethics Committee	Institute Name	Investigator Name	Initial Approval Date
Ulsan University Hospital Institutional Review Board	Ulsan University Hospital, University of Ulsan College of Medicine, Ulsan, Korea	Gyung-Min Park	02-Mar-2023
Chonnam National University Hospital Institutional Review Board for Biomedical Research Ethics	Chonnam National University Medical School, Chonnam National University Hospital, Gwangju, Korea	Young Joon Hong	31-May-2023
Keimyung University Dongsan Hospital Institutional Review Board	Keimyung University, Dongsan Hospital, Daegu, Korea	Chang-Wook Nam	17-Apr-2023
Kyungpook National University Hospital Clinical Trial Review Committee	Kyungpook National University Hospital, Daegu, Korea	Jang Hoon Lee	20-Apr-2023
Seoul National University Bundang Hospital Institutional Bioethics Review Board	Seoul National University Bundang Hospital, Seongnam-si, Korea	In-Ho Chae (Coordinating Investigator)	09-May-2023
Inje University Ilsan Paik Hospital Institutional Review Board for Medical Research Ethics	Inje University College of Medicine, Ilsan Paik Hospital, Goyang, Korea	Sung Uk Kwon	30-Jun-2023
Kangbuk Samsung Hospital Research Ethics Committee	Kangbuk Samsung Hospital, Sungkyunkwan University School of Medicine, Seoul, Korea	Jong-Young Lee	21-Jun-2023
The Catholic University of Korea Eunpyeong St. Mary’s Hospital Clinical Research Review Committee	Eunpyeong St. Mary’s Hospital, College of Medicine, The Catholic University of Korea, Seoul, Korea	Suk Min Seo	11-Jan-2024
