# Supplementary material for: The role of early ezetimibe combination with atorvastatin in patients with atherosclerotic cardiovascular disease
Source: BMC Cardiovasc Disord. 2026 Feb 11;26:233. doi: 10.1186/s12872-026-05594-2 (PMC12998120; doi:10.1186/s12872-026-05594-2)

**The Role of Early Ezetimibe Combination with Atorvastatin in Patients with Atherosclerotic Cardiovascular Disease**

**Supplementary Materials**

**Supplementary Tables and Figures**

**Supplementary Table 1. Baseline LDL-C Summary**

| **Parameters** | **EZ/AS**  **(N=65)** | **AS**  **(N=67)** | **Total**  **(N=132)** |
| --- | --- | --- | --- |
| Baseline LDL-C (mg/dL) | 65 (98.5 ± 24.0) | 67 (108.5 ± 34.4) | 132 (103.6 ± 30.0) |
| History of ≥ 4 weeks of statin  administration | 45 (89.9 ± 14.6) | 41 (95.6 ± 27.7) | 86 (92.6 ± 21.9) |
| History of <4 weeks of statin  administration | 14 (120.9 ± 31.3) | 18 (122.3 ± 34.8) | 32 (121.7 ± 32.8) |
| No history of statin  administration | 6 (111.0 ± 26.6) | 8 (143.8 ± 30.3) | 14 (129.7 ± 32.4) |

Values are Nx (mean ± SD).

N=number of patients in Full Analysis Set; Nx=number of patients without missing values for each variable.

AS=atorvastatin; EZ=ezetimibe; LDL-C=low-density lipoprotein cholesterol; SD=standard deviation.

**Supplementary Table 2. Medical and Treatment History**

| **Statistics** | **EZ/AS (N=65)** | **AS (N=67)** | **Total (N=132)** |
| --- | --- | --- | --- |
| Dyslipidemia duration, mean ± SD years | 4.7 ± 6.4 | 3.7 ± 5.2 | 4.2 ± 5.8 |
| ACS | 38 (58.5) | 39 (58.2) | 77 (58.3) |
| Stable angina | 26 (40.0) | 31 (46.3) | 57 (43.2) |
| Coronary revascularization | 38 (58.5) | 34 (50.7) | 72 (54.5) |
| Stroke | 6 (9.2) | 6 (9.0) | 12 (9.1) |
| Transient ischemic attack | 4 (6.2) | 0 | 4 (3.0) |
| Peripheral arterial disease | 0 | 0 | 0 |
| Other clinical or unequivocal ASCVD on imaging | 3 (4.6) | 2 (3.0) | 5 (3.8) |
| Prior statin therapy, Nx | 65 | 67 | 132 |
| Low- to moderate-intensity statin ≥4 weeks | 45 (69.2) | 41 (61.2) | 86 (65.2) |
| Low- to moderate-intensity statin <4 weeks | 14 (21.5) | 18 (26.9) | 32 (24.2) |
| Statin-naïve, n (%) | 6 (9.2) | 8 (11.9) | 14 (10.6) |

Values are mean ± SD or n (%).

N=number of patients in Full Analysis Set; Nx = number of patients without missing values for each variable; n = number of patients in each category; percentage (%) = 100*(n/Nx).

ACS=acute coronary syndrome; ASCVD=atherosclerotic cardiovascular disease; AS=atorvastatin; EZ=ezetimibe; SD=standard deviation.

**Supplementary Table 3. Extent of Exposure to Study Intervention**

| **Parameters** | **EZ/AS (N=67)** | **AS (N=69)** | **Total (N=136)** |
| --- | --- | --- | --- |
| Treatment Exposure, Mean ± SD days | 78.5 ± 17.7 | 80.6 ± 15.0 | 79.6 ± 16.3 |
| Compliance %, Mean ± SD | 95.9 ± 11.1 | 97.2 ± 10.2 | 96.6 ± 10.6 |
| Compliant, n (%) | 64 (95.5) | 68 (98.6) | 132 (97.1) |

N=number of patients in Safety Analysis Set; n=number of patients in each category; percentage (%) = 100*(n/Nx).

AS=atorvastatin; EZ=ezetimibe; SD=standard deviation.

Note: Treatment exposure (days) is calculated using (date of last dose of study medication – date of first dose of study medication + 1). Total number of tablets taken is calculated as sum of all actual amount administered during the study period. Compliance is calculated using (total number of tablets taken/treatment exposure) x 100. A patient is considered compliant if their percent compliance is at least 80% and no more than 120%.

**Supplementary Table 4. Summary of TEAEs by System Organ Class and Preferred Term**

| **System Organ Class**  **Preferred Term, n (%)** | **EZ/AS (N=67)** | | **AS (N=69)** | **Total (N=136)** |
| --- | --- | --- | --- | --- |
| Any TEAE | 15 (22.4) | | 13 (18.8) | 28 (20.6) |
| Investigations | 5 (7.5) | | 1 (1.4) | 6 (4.4) |
| ALT increased | 3 (4.5) | | 0 | 3 (2.2) |
| AST increased | 2 (3.0) | | 0 | 2 (1.5) |
| GGT increased | 1 (1.5) | | 1 (1.4) | 2 (1.5) |
| AST/ALT ratio abnormal | 1 (1.5) | | 0 | 1 (0.7) |
| Blood ALP increased | 0 | | 1 (1.4) | 1 (0.7) |
| Blood CRPK increased | 1 (1.5) | | 0 | 1 (0.7) |
| General disorders and administration site conditions | 5 (7.5) | | 0 | 5 (3.7) |
| Chest discomfort | 2 (3.0) | | 0 | 2 (1.5) |
| Fatigue | 1 (1.5) | | 0 | 1 (0.7) |
| Musculoskeletal and connective tissue disorders | | 0 | 4 (5.8) | 4 (2.9) |
| Myalgia | | 0 | 3 (4.3) | 3 (2.2) |
| Nervous system disorders | | 1 (1.5) | 3 (4.3) | 4 (2.9) |
| Dizziness | | 1 (1.5) | 2 (2.9) | 3 (2.2) |
| Cerebral hemorrhage | | 0 | 1 (1.4) | 1 (0.7) |
| Blood and lymphatic system disorders | | 2 (3.0) | 1 (1.4) | 3 (2.2) |
| Anemia | | 1 (1.5) | 1 (1.4) | 2 (1.5) |
| Gastrointestinal disorders | | 1 (1.5) | 2 (2.9) | 3 (2.2) |
| Hematochezia | | 0 | 1 (1.4) | 1 (0.7) |
| Melaena | | 1 (1.5) | 0 | 1 (0.7) |
| Metabolism and nutrition disorders | | 0 | 2 (2.9) | 2 (1.5) |
| Neoplasms benign, malignant and unspecified | | 0 | 2 (2.9) | 2 (1.5) |
| Adenocarcinoma | | 0 | 1 (1.4) | 1 (0.7) |
| Gastric cancer | | 0 | 1 (1.4) | 1 (0.7) |
| Respiratory, thoracic and mediastinal disorders | | 2 (3.0) | 0 | 2 (1.5) |
| Dyspnea | | 1 (1.5) | 0 | 1 (0.7) |
| Skin and subcutaneous tissue disorders | | 2 (3.0) | 0 | 2 (1.5) |
| Acute MI | | 1 (1.5) | 0 | 1 (0.7) |
| Vascular disorders | | 0 | 1 (1.4) | 1 (0.7) |

n=number of patients in each category; percentage (%) = 100 * (n / N).

ALP=alkaline phosphatase; AS=atorvastatin; AST/ALT=aspartate transaminase/alanine transaminase; CRPK=creatine phosphokinase; EZ=ezetimibe; GGT=Gamma-glutamyl transferase; MI=myocardial infarction; TEAE=treatment-emergent adverse event. N=number of patients in Safety Analysis Set.

**Supplementary Figure 1. Study Design**


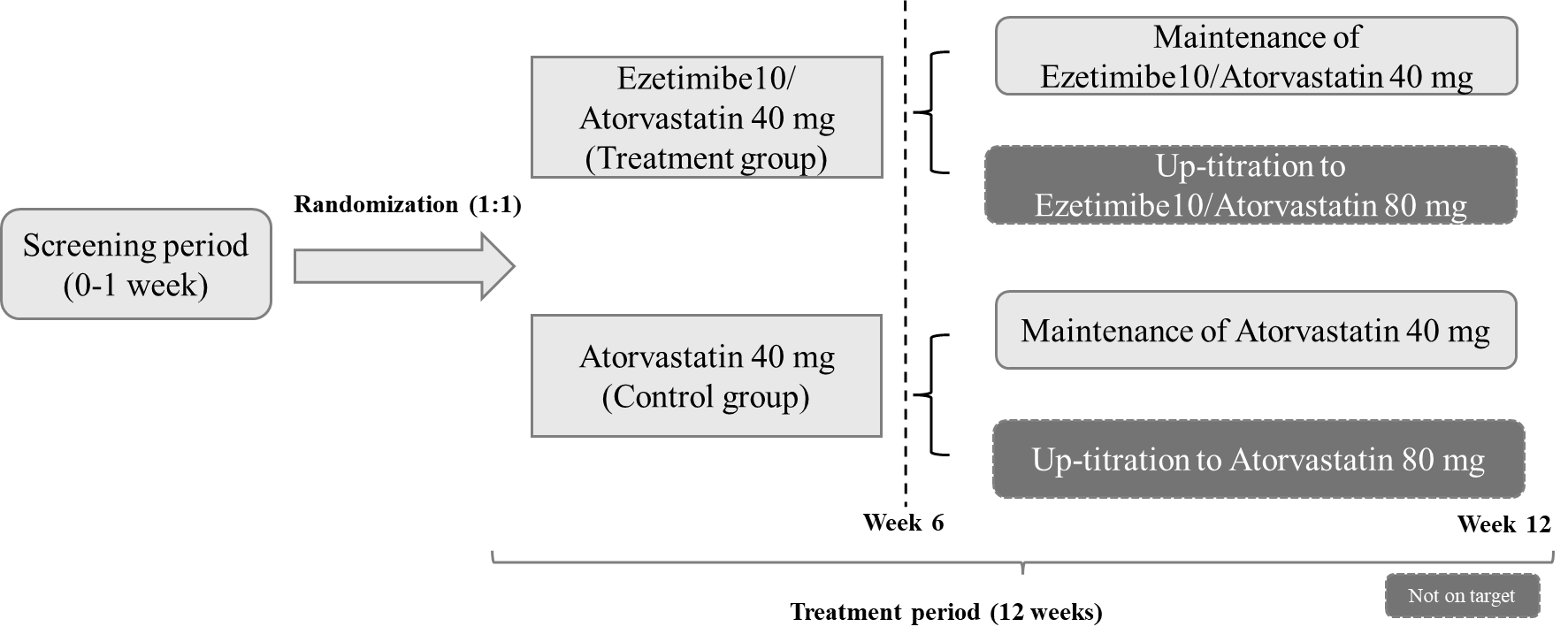

Supplement: Supplementary file 1 — Supplementary Material 1. [file 12872_2026_5594_MOESM1_ESM.docx]
